# The HSP70 Molecular Chaperone Is Not Beneficial in a Mouse Model of α-synucleinopathy

**DOI:** 10.1371/journal.pone.0010014

**Published:** 2010-04-02

**Authors:** Derya R. Shimshek, Matthias Mueller, Christoph Wiessner, Tatjana Schweizer, P. Herman van der Putten

**Affiliations:** 1 Neuroscience Research, Novartis Institutes for BioMedical Research, Novartis Pharma AG, Basel, Switzerland; 2 Developmental and Molecular Pathways/Model Disease Centre, Novartis Institutes for BioMedical Research, Novartis Pharma AG, Basel, Switzerland; National Institutes of Health, United States of America

## Abstract

**Background:**

Aggregation and misfolded α-synuclein is thought to be central in the pathogenesis of Parkinson's disease (PD). Heat-shock proteins (HSPs) that are involved in refolding and degradation processes could lower the aggregate load of α-synuclein and thus be beneficial in α-synucleinopathies.

**Methodology/Principal Findings:**

We co-overexpressed human A53T point-mutated α-synuclein and human HSP70 in mice, both under the control of Thy1 regulatory sequences. Behavior read-outs showed no beneficial effect of HSP70 expression in mice. In contrast, motor coordination, grip strength and weight were even worse in the α-synucleinopathy model in the presence of HSP70 overexpression. Biochemical analyses revealed no differences in α-synuclein oligomers/aggregates, truncations and phosphorylation levels and α-synuclein localization was unchanged in immunostainings.

**Conclusion/Significance:**

Overexpressing HSP70 in a mouse model of α-synucleinopathy did not lower the toxic load of α-synuclein species and had no beneficial effect on α-synuclein-related motor deficits.

## Introduction

Mutations in the α-synuclein gene and multiplications of the α-synuclein locus have been linked to familiar forms of PD [1]–[6]. In addition, Lewy bodies, the pathological hallmark of sporadic PD, contain aggregates of α-synuclein and multiple additional components like ubiquitin [7], [8]. α-synuclein is natively unfolded and can self-aggregate to form oligomers and fibrils which are believed to be involved in the pathogenesis of PD. Up-to-date, it is unclear how and to what extent aggregates of α-synuclein influences neuronal dysfunction and subsequently leads to neurodegeneration. However, it is widely accepted that decreasing α-synuclein oligomers, fibrils and aggregates will positively affect α-synuclein-induced toxicity [9].

Heat shock proteins (HSPs) belong to the family of chaperone proteins and are important in refolding misfolded proteins, in preventing protein aggregate formation and targeting proteins to proteasomal degradation [10], [11]. It has been shown that HSPs can be protective in several neurodegenerative models [12]–[15]. In particular, HSP70 inhibits α-synuclein fibril assembly [16], [17], protects against α-synuclein toxicity *in vitro* [17]–[19] and reduces α-synuclein aggregates *in vivo* [18]. We tested this hypothesis using the mouse model of α-synucleinopathy that overexpresses human α-synuclein containing the A53T point-mutation [20] and co-overexpress HSP70, both under the control of the Thy1 promoter. We demonstrate here that HSP70 has no beneficial effect on behavior and α-synuclein aggregation *in vivo*.

## Results and Discussion

Several studies report beneficial effects of HSPs, especially that of HSP70 on α-synuclein aggregation and toxicity *in vitro* [16]–[19], [21] and *in vivo* [12], [18]. HSPs function as molecular chaperones; they are engaged in folding and refolding processes, protein transport and in protein degradation. We tested the hypothesis that HSP70 reduces the toxic load of α-synuclein *in vivo*.

**Figure 1:**
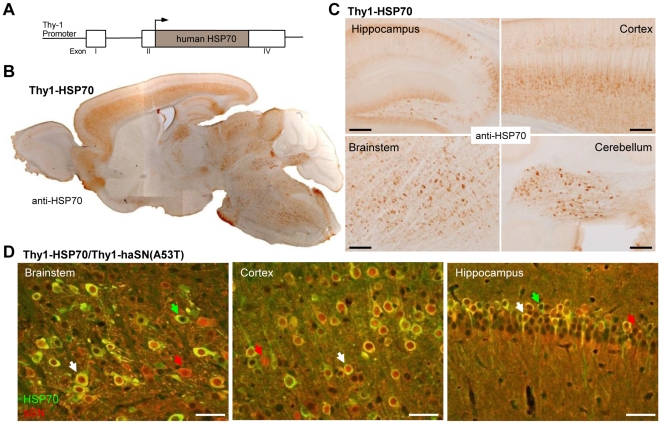
Mice overexpressing human HSP70 under the control of Thy1 regulatory sequences. A. Schematic diagram of the Thy1-HSP70 construct. B. Overview section of a Thy1-HSP70 mouse brain stained against HSP70. C. Details of brain sections from Thy1-HSP70 mice showing HSP70 expression in different brain regions. Scale bars: 200 µm. D. Co-localization of human HSP70 (green) and human α-synuclein (red) in different brain regions of double transgenic Thy1-HSP70/Thy1-haSN(A53T) mice. White arrow: Co-localization of HSP70 and α-synuclein; Red arrow: neurons expressing only human α-synuclein; Green arrow: neurons single positive for human HSP70. Scale bars: 50 µm.

We generated mice expressing human HSP70 (HSPA1A) under the control of Thy1 regulatory sequences (Figure 1A). Five founders were obtained and HSP70 expression was analysed by immunohistology whereas one line had stable and high HSP70 expression (Figure 1B and 1C, named Thy1-HSP70 hereafter). The transgene expression was highest in cortical, hippocampal and brainstem areas, whereas it was low in the olfactory bulb and cerebellum and absent in striatum. Thus, the expression pattern reflects that of the Thy1 promoter observed before in other transgenic mice utilizing the same promoter [20]. The Thy1-HSP70 line was crossbred with Thy1-9813 (named Thy1-haSN(A53T) hereafter) described earlier [20]. Thy1-haSN(A53T) mice display severe motor coordination deficits and develop subsequently neuropathology in several brain areas and degeneration of the neuromuscular junctions. The resulting double transgenic mice Thy1-HSP70/Thy1-haSN(A53T) showed a high proportion of neuronal co-localization of HSP70 and α-synuclein in different brain areas (Figure 1D). Only a few neurons were single-positive for HSP70 or α-synuclein. This good overlap of expression pattern makes these double transgenic animals suitable for analyzing a beneficial effect of HSP70 on α-synucleinopathy.

**Figure 2:**
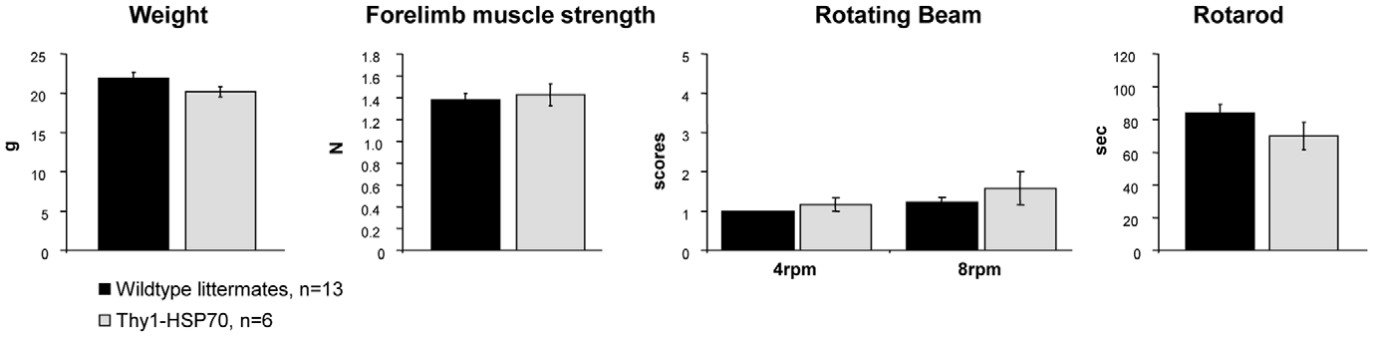
Thy1-HSP70 mice appear and behave normally and are phenotypical indifferent to wildtype mice. Measurement of weight, forelimb grip strength and motor coordination (rotating beam and rotarod) of single Thy1-HSP70 and wildtype mice. Error bars: SEM.

The overexpression of HSP70 throughout the brain did not alter behavior in Thy1-HSP70 compared to wild-type littermates (Figure 2). Weight and forelimb muscle strength did not differ as well as motor coordination tested by two different tasks. Thus, these two groups were pooled (named control) for statistical analyses.

**Figure 3:**
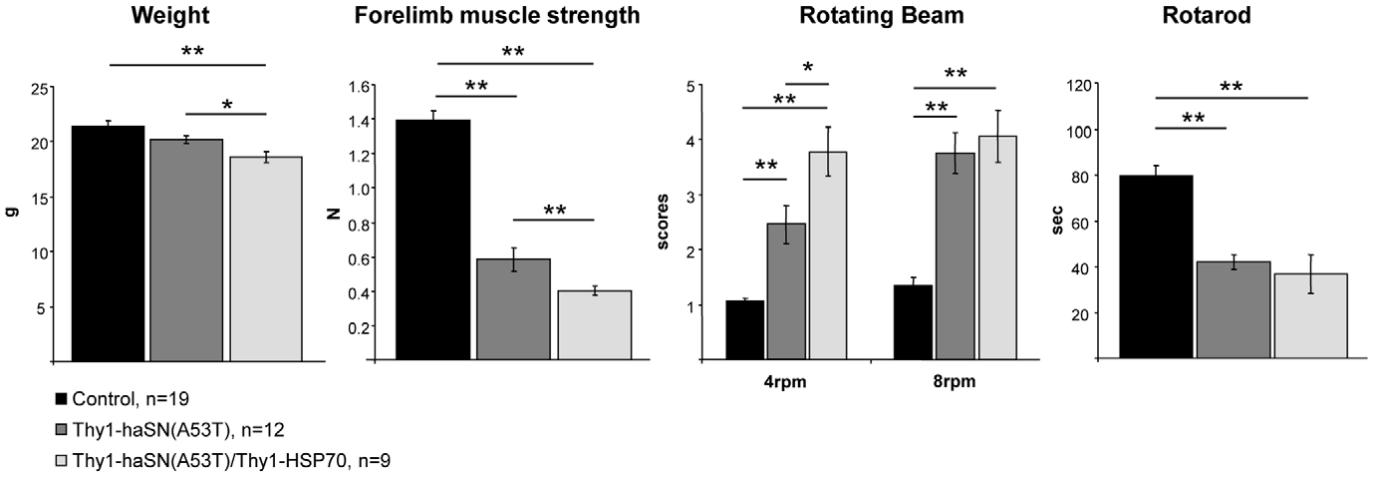
HSP70 overexpression in an α-synucleinopathy mouse model is not beneficial but even worsens the phenotype. Measurement of weight, forelimb grip strength and motor coordination (rotating beam and rotarod) of single- and double-transgenic Thy1-HSP70/Thy1-haSN(A53T) mice. Asteriks indicate statistical significance (*: p<0.05, **: p<0.01; student's t-test, two-tailed). Error bars: SEM.

Thy1-haSN(A53T) mice showed at four months of age severe motor coordination deficits (Figure 3 and [20]) and in addition showed extensive muscle weakness. Weight was normal. However, double transgenic Thy1-HSP70/Thy1-haSN(A53T) mice (age: 4 months) were even more impaired in their motor behavior (see Figure 3, rotating beam at 4 rpm), had a more pronounced forelimb muscle weakness and a significant reduction of weight compared to single-positive Thy1-haSN(A53T) mice (Figure 3). Mice were killed between six and seven months of age. It is very difficult to assess histopathology in Thy1-haSN(A53T) mice as the age of onset to manifest neuropathology is very heterogeneous and hence, the quantification substantially variable. Thus, we focused on the protein biochemistry of α-synuclein to analyse α-synuclein oligomers and aggregates from these animals.

**Figure 4:**
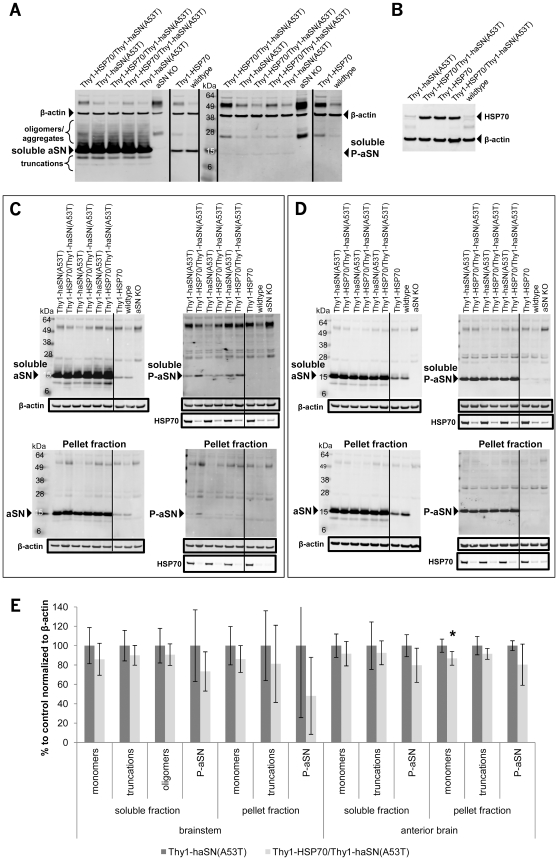
Biochemical analyses of soluble and pellet fraction protein extracts from spinal cord, brainstem and anterior brain reveal no difference in α-synuclein species. A. Immunoblot of mouse spinal cord lysates detecting soluble α-synuclein monomers, oligomers/aggregates, truncations and S129-phosphorylation (P-aSN). B. Immunoblot of mouse spinal cord lysates detecting soluble HSP70. C. Immunoblot of soluble (supernatant) and pellet fractions of brainstem detecting HSP70 and α-synuclein monomers, oligomers/aggregates, truncations and S129-phosphorylation. D. Immunoblot of soluble (supernatant) and pellet fractions of anterior brain detecting HSP70 and α-synuclein monomers, truncations and S129-phosphorylation (P-aSN). β-actin was used as loading control. The size markers are indicated. E. Bar graphs representing quantitative analyses of the immunoblots shown in C and D for the α-synuclein monomers, oligomers/aggregates, truncated forms and S129-phosphorylation (P-aSN) normalized to the loading control β-actin in soluble and pellet fractions. n = 5 per genotype. Asteriks indicate statistical significance (*: p<0.05); student's t-test, two-tailed. Error bars: standard deviation.

Protein extracts from spinal cord, brainstem and anterior brain (lacking cerebellum and brainstem) from single and double transgenic mice were analysed in immunoblots for HSP70 and α-synuclein. Homogenates without detergent were generated and soluble (supernatant after 13k rpm centrifugation) and pellet (resuspension of crude pellet in detergent-free buffer after centrifugation and removal of supernatant) fractions were collected. As a negative control α-synuclein knock-out mice were used. In soluble fractions from spinal cord and brainstem, Thy1-haSN(A53T) showed a high overexpression of α-synuclein and beside the α-synuclein monomer multiple bands for α-synuclein were visible indicating the existence of α-synuclein oligomers/aggregates (Figure 4A and 4C). Interestingly, α-synuclein oligomers/aggregates could not be detected in pellet fractions from brainstem (Figure 4C), spinal cord (data not shown) and in soluble extracts from anterior brain (Figure 4D). Two truncated forms of α-synuclein were detected in spinal cord and brainstem that are absent in wild-type and α-synuclein knock-out (Figure 4A and 4C) and somewhat weaker in pellet fractions of brainstem (Figure 4C) and in anterior brain (Figure 4D). S129-phosphorylated α-synuclein was, interestingly, very high in anterior brain in soluble and pellet fractions (Figure 4D) and weak in spinal cord and brainstem (Figure 4A and 4C). Higher molecular weight species for α-synuclein (trimers, tetramers, etc.) could not be observed as described elsewhere [9]. This could be because of a different mouse line used or a different antibody recognizing another epitope. In addition, α-synuclein oligomers/aggregates are not phosphorylated at serine 129. HSP70 protein can be detected in soluble and pellet fractions and seems not to be influenced by α-synuclein (Figure 4). Qualitative (spinal cord (Figure 4A); single (n = 5–6) and double transgenic (n = 11) on different immunoblots) and quantitative analysis of α-synuclein oligomers/aggregates, truncations and phosphorylation in soluble and pellet fractions of brainstem and anterior brain (Figure 4E) (n = 5, each for single and double transgenic mice) revealed no significant change on α-synuclein oligomer/aggregated species, truncations and S129-phosphorylation in Thy1-HSP70/Thy1-haSN(A53T) double transgenic animals despite a strong HSP70 expression (Figure 4B, 4C and 4D). Brainstem samples somehow show a greater variation in phosphorylated α-synuclein and in the analysis of the pellet fraction compared to those from anterior brain, although β-actin and HSP70 levels are relatively constant. Only for α-synuclein monomers in the pellet fraction of anterior brain could a small but significant reduction of 13% be observed. This finding should be taken cautiously as our experience point to a pure variability of the mouse line and/or of the pellet fraction analyses. As no other parameter is significantly changed and, more importantly, α-synuclein oligomers/aggregates are not affected by the presence of HSP70 our conclusion is that HSP70 has no effect on α-synuclein load. Our data does not support the hypothesis that enhanced chaperone protein function by HSP70 can alter α-synuclein misfolding *in vivo*. Although, a possible explanation for a lack of chaperone effect on α-synuclein metabolism could be due to a non-functional or only partially functional HSP70 as *in vivo* functionality of the overexpressed human HSP70 in mice has not been determined due to several experimental challenges. Nevertheless, Thy1-HSP70/Thy1-haSN(A53T) double transgenic animals were even worse in motor behavior than their single transgenic Thy1-haSN(A53T) littermates. Several studies, however, claim that solely by the presence of the chaperone HSP70 α-synuclein oligomer formation could be prevented [16]–[19]. In contrast, others showed only positive effects on α-synuclein fibers with HSP104 and not with HSP70 [22].

**Figure 5:**
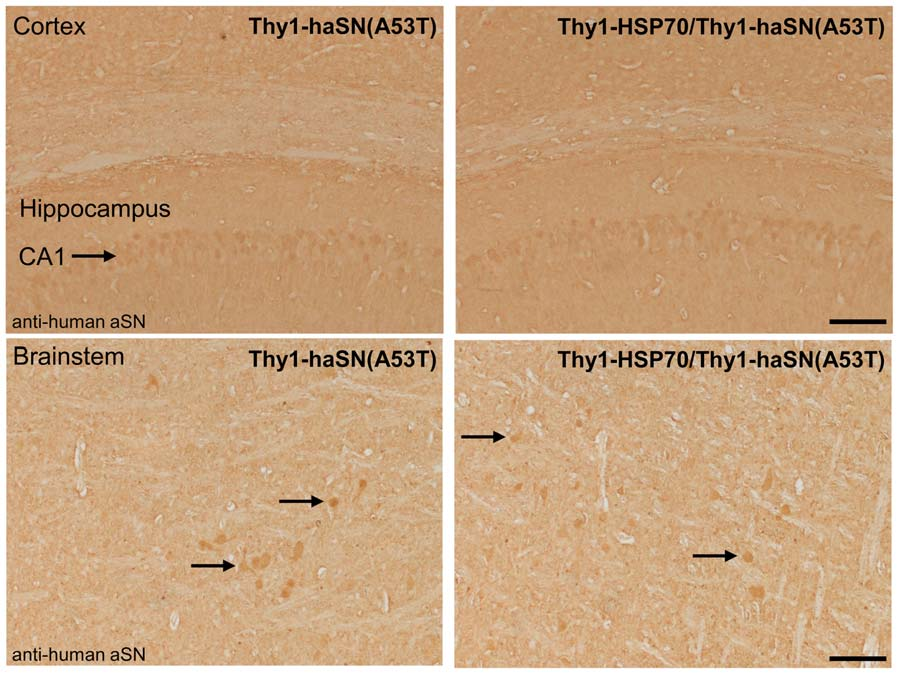
Histological analyses reveal no difference in α-synuclein distribution/localization in the brain. Brain sections of single and double transgenic Thy1-HSP70/Thy1-haSN(A53T) mice stained against human α-synuclein. Black arrow: cytoplasmic and nuclear α-synuclein in the CA1 area of the hippocampus and brainstem neurons. Scale bar: 100 µm.

α-synuclein is normally synapticaly localized, however, neurons with high overexpression of α-synuclein reveal a strong cytoplamic and nuclear staining of mislocalized α-synuclein as seen in Thy1-haSN(A53T) mice (see red arrows in Figure 1D). To evaluate if excessive HSP70 expression could rescue this α-synuclein mislocalization despite unchanged protein levels/oligomers we looked in detail to the distribution of α-synuclein in Thy1-HSP70/Thy1-haSN(A53T) mouse brains. There was no difference detectable between Thy1-haSN(A53T) single-positive and Thy1-HSP70/Thy1-haSN(A53T) double transgenic animals. Somatic/nuclear α-synuclein localization was unchanged in different brain areas (Figure 1D and 5).

Interestingly, α-synuclein overexpression in single transgenic Thy1-haSN(A53T) mice did not induce the expression of HSP70, and Thy1-HSP70 mice did not show any change in endogenous α-synclein levels (Figure 4). One would suggest that α-synuclein overexpression is a stress factor to cells and as a consequence would activate the heat-shock-response, amongst others HSP70. However, we have not analysed the expression of other HSPs.

It has been reported that HSP27 but not HSP70 has a protective effect on α-synuclein-induced toxicity [17], [23] as well as HSP104 in a rat model of Parkinson disease [22]. In addition, HSP70 was not very effective in a polyglutamine mouse model [24]. However, in toxin-induced mouse models HSP70 overexpression was protective [13]. Thus, other HSPs are needed besides HSP70 to achieve clear positive effects on α-synucleinopathies.

In summary, our results demonstrate that HSP70 alone is not sufficient to alter behavior deficits and α-synuclein species in a mouse model of α-synucleinopathy.

## Materials and Methods

### Statement on Animal Welfare

All experiments were carried out in accordance with authorization guidelines for the care and use of laboratory animals. Studies described in this report were performed according to Novartis animal license number 2063.

### Transgenic Mice

Heat shock protein 70kDa 1A (HSPA1A) cDNA (1926 bp) was cloned into the Thy1 cassette (van der Putten et al., 2000) and transgenic Thy1-HSP70 mice were generated by pronuclear injection (C57Bl/6 mouse eggs) of linearized (NotI) and purified minigene. Transgenic mice were selected by PCR analysis of tail DNA with primers Thy1for2 (5′-GGGCTGACCTGGACATTAGG-3′) and HSP70rev2 (5′-GTTCAGCGCCACCTGGTT CT-3′), which amplified with a standard PCR protocol a 991 bp DNA fragment. Thy1-haSN(A53T) (van der Putten et al., 2000) were genotyped by using primers HP45 (ACACCCCTAAAGCATACAGTCAGACC) and HP42 (TGGGCACATTGGAACTGA GCACTT), amplified DNA fragment: 1200 bp. Double transgenic mice Thy1-HSP70/Thy1-haSN(A53T) were identified by PCR analysis of tail DNA using two independent rounds of PCR, one for Thy1-HSP70 and Thy1-haSN(A53T), respectively. Trangenic mice were kept in C57Bl/6 background. For analyses males and females were used.

### Rotating Beam

The rotating beam was build in-house and is connected to a rotarod apparatus (Lugo Basile, Italy). It consists of a metal beam (Ø 1 cm, length 122 cm) coated with rubber attached to a rotarod apparatus (gradient angle upwards of 10°) which controls the constant rotating speed (4 rpm and 8 rpm). The beam is divided into four equal sections which are used for scoring the performance of the mice (scores 1–5). The mouse is placed at the beginning on the already rotating beam facing upwards. Score 1 (best): mouse reaches the end of the beam without falling down or hanging on the beam head-down; Score 2: mouse falls down or is hanging head-down in the section 4 of beam; Score 3: mouse falls down or is hanging head-down in the section 3 of beam; Score 4: mouse falls down or is hanging head-down in the section 2 of beam; Score 5: mouse falls down or is hanging head-down in the section 1 of beam. Trials are finished, if a mouse reaches the end of the beam, hangs at the beam head-down, or falls down completely. One session consists out of two trials (each max. 1 min) of 4 rpm and two trials of 8 rpm, whereas the sequence is one trial of 4 rpm followed by on trial of 8 rpm in the morning and in the afternoon.

### Rotarod

To measure motor coordination mice were placed on a computerized treadmill (TSE rotarod system, Germany) and the latency to fall-off the rotating rod is determined. The rotarod program consists of three different running speeds (12 rpm, 24 rpm and 36 rpm) each for 30 sec with intervals of acceleration lasting for 10 sec. Starting speed is 4 rpm. Rotarod performance was assessed by evaluating the two best trials out of three performed in one day.

### Forelimb grip strength

To measure forelimb grip strength, mice are allowed to grasp a handle connected to a force-measuring device (San Diego Instruments, USA) and then pulled back with their tails until they release the handle. The best out of four consecutive trials is evaluated in N.

### Biochemistry

All work was carried out at 4°C. Mouse brain was added to 5ml homogenization buffer (20mM Tris-HCl, pH 7.4, 0.25M sucrose, 1mM EDTA, 1mM EGTA, 0.5mM PMSF, 0.5µg/ml pepstatin A and leupeptin) without any detergent, then homogenized and centrifuged in an Eppendorf centrifuge 5415R at 13000 rpm for 20 minutes at 4°C. The protein concentration of the supernatant was determined using a protein assay kit (Bio-Rad Laboratories, CA, USA). For immunoblot analysis, 10µg/slot of supernatant (treated with NuPAGE LDS sample buffer (4×) and NuPAGE sample reducing agent (10×) at 95°C for 5 min) was analysed by PAGE (NuPAGE Novex Bis-Tris gels (4–12%), Invitrogen, CA, USA). The pellet fraction was dissolved in the same homogenization buffer as above without detergent (10× volume of the tissue weight) and then treated like and used according to supernatant samples. For blotting XCell II blot module (Invitrogen, CA, USA) was used. Detection was performed using the following antibodies: Mouse monoclonal antibodies α-synuclein (1∶500, BD Biosciences, CA, USA), phosphorylated α-synuclein (1∶5000, WAKO, Japan), HSP70 (1∶5000, StressGen, MI, USA) and as secondary antibody the F(ab')2 fragment of goat anti-mouse IgG, labeled with Alexa Fluor 680 (1∶2000, Invitrogen, CA, USA). The LI-COR Odyssey System (LI-COR Biosciences GmbH, Germany) was used for quantification of band intensities as described by the supplier. Fluorescence intensities quantification was performed by standardization to β-actin levels using a mouse monoclonal anti β-actin clone AC-15 antibody (1∶5000, Sigma Aldrich, Switzerland).

### Immunohistochemistry

PBS-perfused brains were postfixed overnight in 4% formalin in PBS at 4°C. For paraffin sections, the brains were dehydrated by different ethanol treatments using a Tissue-Tek VIP system (GMI Inc, MI, USA). The ethanol treatment series consisted of 70%, 80%, 90%, 2×94%, 3×100% ethanol each for 1h. Brains were then washed 2 times in xylol for 30 min followed by 2 times in paraffin (30 min, 60 min). Subsequent, the dehydrated brains were embedded at 55°C in paraffin using a Tissue Block System TBS 88 (Geneq Inc, Canada). Slices were cut on a Microm HM 355 device (Microm International GmbH, Germany) at a thickness of 4–5 µm and mounted on superfrost glass slides (Microm International GmbH, Germany) and air-dried. Slides were kept at room temperature until use. Paraffin was removed by treating the slides with xylene (2×10 min) followed by an ethanol treatment series of 2×100%, 2×95% and 1×70% (2–3 min each). Slides were then rinsed 5 min in distilled water. For immunohistochemistry of free-floating slices 50 µm thick vibratome sections were cut of the post-fixed brains. Slides were incubated 3×10 min in day-1 buffer (0.01 M PBS, 1% BSA, 0.3% Triton X-100) and blocked by addition of 4% normal goat serum for 20 min. Paraffin-embedded slides were incubated in day-1 buffer with 1% normal goat serum and subsequently incubated overnight with a mouse monoclonal anti-HSP70 (SPA-810, 1∶200, StressGen, MI, USA) and aSN antibody (1∶800, 4B12, Abcam, UK) in a humidity chamber at room temperature. Free-floating sections were incubated with HSP70 antibody (SPA-810, 1∶800, StressGen, MI, USA) and α-synuclein mouse monoclonal antibody (Syn-1, 1∶500; S63320, Transduction Laboratories, Lexington, KY). Slides or sections were then rinsed 3×10 min in day-2 buffer (day-1 buffer diluted 1∶3 in 0.01M PBS). Slides or sections were incubated for 1 h with secondary antibody (Biotin-conjugated goat anti-mouse IgG (1∶200, Vectorlabs, CA, USA) and then with ABC reagent (Vectorlabs, CA, USA) for 1 h at room temperature. Slides or sections were then rinsed in day-2 buffer 2×10 min, in 0.01 M PBS 2×20 min and were then desalted in 0.01M Tris (pH 7.8). Free-floating brain sections were mounted on glass slides. Slides were air-dried at room temperature and coversliped with eukitt mounting medium and analysed on a Nikon microscope.

### Maintenance

The animals were housed in a temperature-controlled room that was maintained on a 12 hr light/dark cycle. Food and water were available *ad libitum*.
